# Ontogeny of the digestive enzyme activity of the pikeperch (*Sander lucioperca*) under culture condition

**DOI:** 10.1038/s41598-023-43845-w

**Published:** 2023-11-13

**Authors:** Fatemeh Lavajoo, Bahram Falahatkar, Irene García-Meilán, Miquel Perelló-Amorós, Iraj Efatpanah, Joaquim Gutierrez

**Affiliations:** 1 Fisheries Department, Faculty of Natural Resources, University of Guilan, Sowmeh Sara, Guilan Iran; 2 Department of Cell Biology, Physiology and Immunology, Faculty of Biology, University of Barcelona, Barcelona, Spain

**Keywords:** Biochemistry, Developmental biology, Physiology

## Abstract

The pikeperch (*Sander lucioperca*) is a species with a high potential for aquaculture and a valuable food with high market acceptance. The aim of the study was to evaluate the functional ontogeny of digestive enzyme of pikeperch from hatching to 45 days-post fertilization, 777 degree-day (DPF, dd) under culture condition. The average total length (TL) of larvae measured at hatching was 3.6 ± 0.4 mm (5 DPF; 67 dd) and at the end of experiment (45 DPF, 777 dd) was 27.1 ± 1.1 mm. The survival rate was 80–90% during the experiment period. Inhibition zimography reveals the presence of nine bands with proteolytic activity in the digestive tract of juvenile pikeperch. Zimography results during the ontogeny revealed that in larvae at 8 DPF (108 dd) and 13 DPF (189 dd), three bands were presented. The variations observed in the enzymatic activity reflected a high amount of total protease activity at 10 DPF (133.5 dd). Regarding pepsin, its activity was observed for the first time at 26 DPF (378.9 dd). Lipase activity remained constant from hatching to 26 DPF (378.9 dd). The highest amount of α-amylase activity was detected at 15 DPF (211.5 dd) and 45 DPF (777 dd). The low lipase enzyme activity suggested that live feeds with low lipid were more suitable than diets containing high lipid levels; larvae had also early capability to digest nutrient-dense diet that was high in protein. According to results the pikeperch larvae possess after the exogenous feeding, a functional digestive system with high activities that indicated the gradual development of the digestive system.

## Introduction

Digestive enzyme study is the first stage of describing and refining the nutritional status during fish ontogeny^1^. The digestive system of fish larvae in the early life stages is incomplete and consists of a straight intestine and absence of stomach function^2^. According to Rønnestad et al.^3^ and Yúfera et al.^4^, feeding activity and utilization of exogenous enzyme of fish larvae are dependent on digestive system function, in which pancreatic enzymes are responsible of the digestion in the early fish stage^2^. The nutritional transition between endogenous (yolk sac reabsorbance) and exogenous feeding is a sensitive period in larviculture, which is considered as a key challenge in feeding protocols adaption with digestive capacities during development^5,6^.

The patterns of digestive enzymes activity are different during fish ontogeny and also among fish larvae and are essential for understanding fish nutritional physiology^5,7^. This changing in digestive enzyme capacities identify the digest diets which can be used and absorbed during the development^3,6,8,9^. So for the larvae survival, it is necessary to fed with digestible food after the depletion of endogenous sources^7^.

The pikeperch *Sander lucioperca* as a member of percidae family is commercially valuable species and is one of the high demand percid species in market capitalization^10^. The fast-growing trait of pikeperch compare to other percids and its great potential in aquaculture make it a notable species for mass cultivation for commercial purposes. Hence, the breeding and rearing of pikeperch increases in many European countries such as Denmark, Czech Republic, Poland, Romania, Hungary, Tunisia, Ukraine and Netherlands^11^. Consequently, the knowledge of the functional digestive system can help to improve the fish diet optimization and feeding strategies during ontogenetic development^10^. In this respect, in the last decades, some studies have focused on the pikeperch larval digestive system and its functional development. Mani-Ponset et al.^12^ studied the development of digestive system in pikeperch larvae according to the first diet. Moreover, the changes in digestive tract of pikeperch larvae fed formulated diets was tested by Ostaszewska et al.^13^. However, the effects of precocious weaning and diets on ontogeny of digestive activities have been documented in this species^10^. After that, Kamaszewski et al.^14^ evaluated the effect of feeding on liver and pancreas and activity of some digestive enzymes in pikeperch, and recently the effect of different feeding regimes with rotifers (*Brachionus plicatilis*) and *Artemia salina* on gene expression and digestive enzymes in pikeperch larvae highlighted by Imentai et al.^15^. Hence, no study was carried out on the ontogeny of the digestive enzyme activity of the pikeperch from hatching to juvenile stages. Our work investigated the functional ontogeny of digestive enzyme of pikeperch from hatching through 40th days post hatching (DPH) under culture condition.

## Results

**Figure 1:**
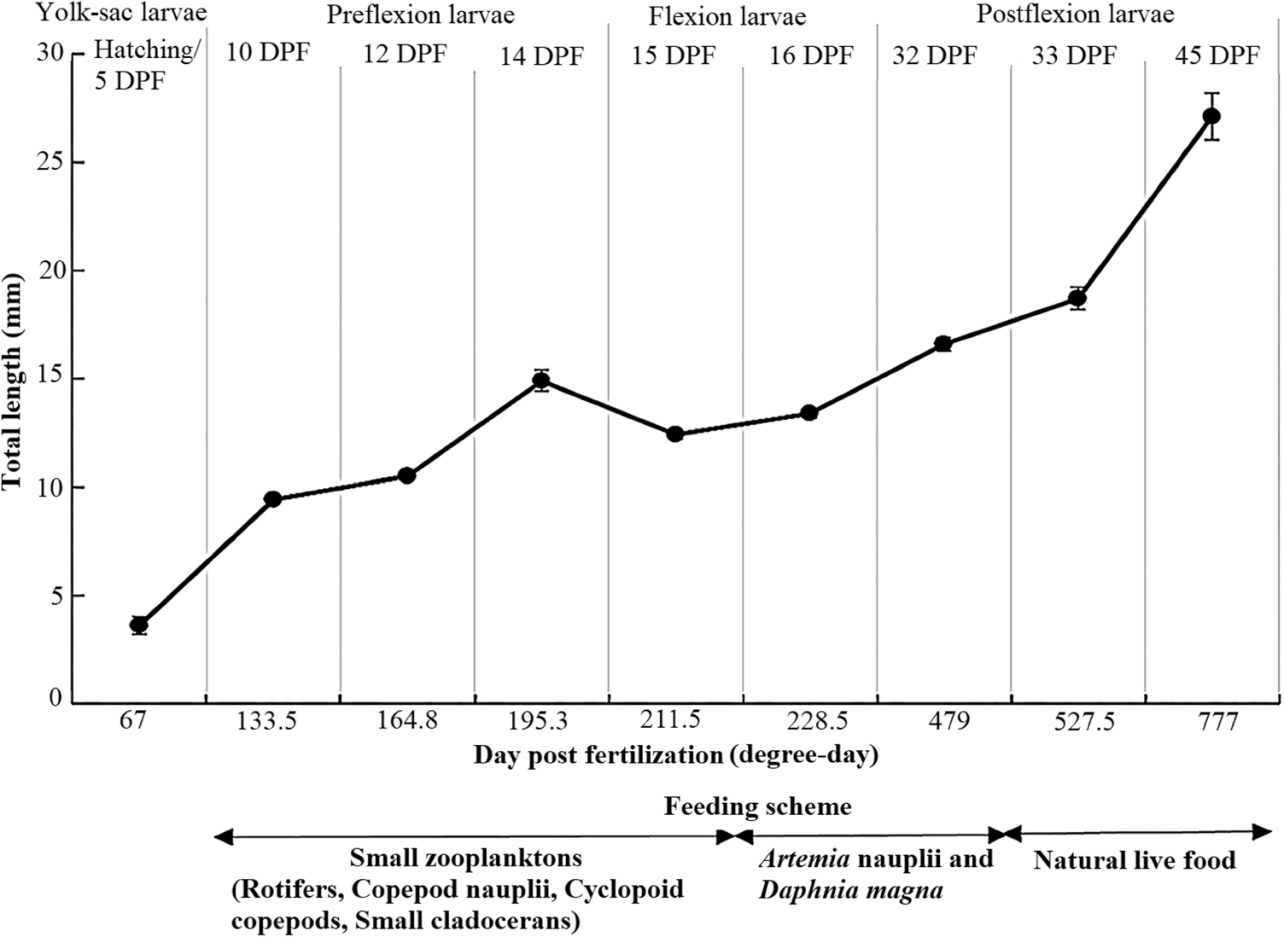
The growth of pikeperch (*Sander lucioperca*) and water temperature fluctuations during the first 45 days post fertilization.

From hatching (5 days post fertilization; 67 degree-day) to the final period of development (45 DPF, 777 dd), the TL of the pikeperch grew continuously and increased from 3.6 ± 0.4 mm to 27.0 ± 1.1 mm, respectively (Fig. 1). The survival rate was 80–90% during the experiment period.

**Figure 2:**
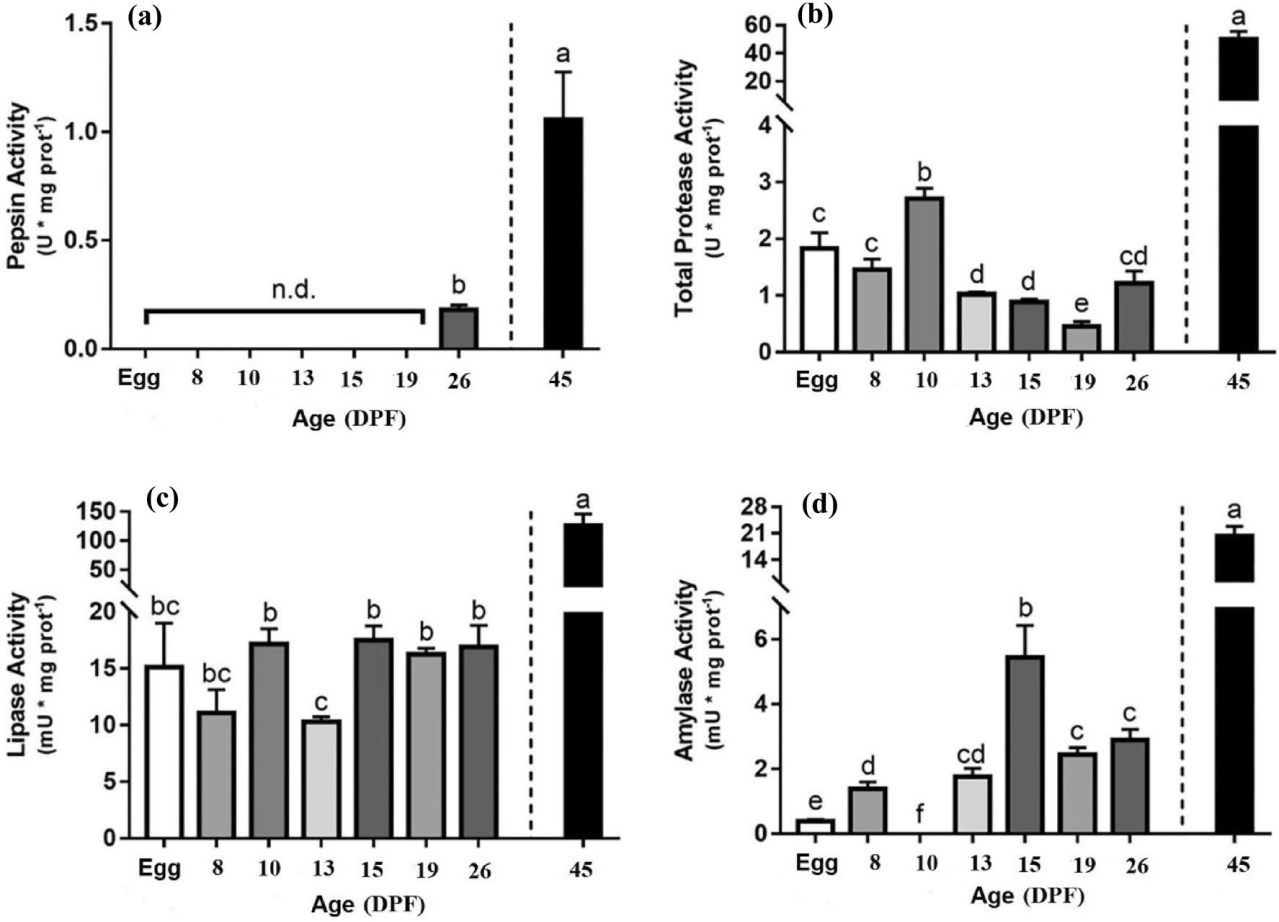
Digestive enzymes activity during ontogeny of pikeperch* Sander lucioperca.* (**a**) pepsin, (**b**) total protease, (**c**) lipase and (**d**) α-amylase activities. Values are presented as mean ± SEM, n = 4 from egg to 26 DPF and n = 9 for juveniles at 45 DPF. Letters show significant differences between groups (*p* < 0.05). DPF: day post fertilization.

Digestive enzyme activities during pikeperch ontogeny from egg to juvenile stage (45 DPF, 777 dd) is presented in Fig. 2. Pepsin activity was observed for the first time at 26 DPF (378.9 dd; 0.18 ± 0.02 U mg protein^−1^) then it increased to maximum level at 45 DPF (777 dd; 1.05 ± 0.21 U mg protein^−1^) (Fig. 2a). According to the results, significant changes were detected in alkaline protease activity during ontogeny (*p* < 0.05). A high amount of alkaline protease activity was detected at 10 DPF (133.5 dd; 2.71 ± 0.18 U mg protein^−1^) then sharply increased at 45 DPF (777 dd; 50.19 ± 5.46 U mg protein^−1^) (Fig. 2b). Differences in lipase activity during ontogeny were significant (*p* < 0.05). Lipase activity remained constant from hatching to 26 DPF (378.9 dd; 14.89 ± 1.6 mU mg protein^−1^), then increased to highest level at 45 DPF (777 dd; 125.69 ± 20.32 mU mg protein^−1^) (Fig. 2c). α-amylase activity was detected from hatching to 45 DPF (777 dd), and it showed significantly differences during larval development (*p* < 0.05), the highest amount was detected on 15 DPF (211.5 dd; 5.45 ± 0.98 mU mg protein^−1^) and 45 DPF (777 dd; 20.40 ± 2.41 mU mg protein^−1^) (Fig. 2d).

**Figure 3:**
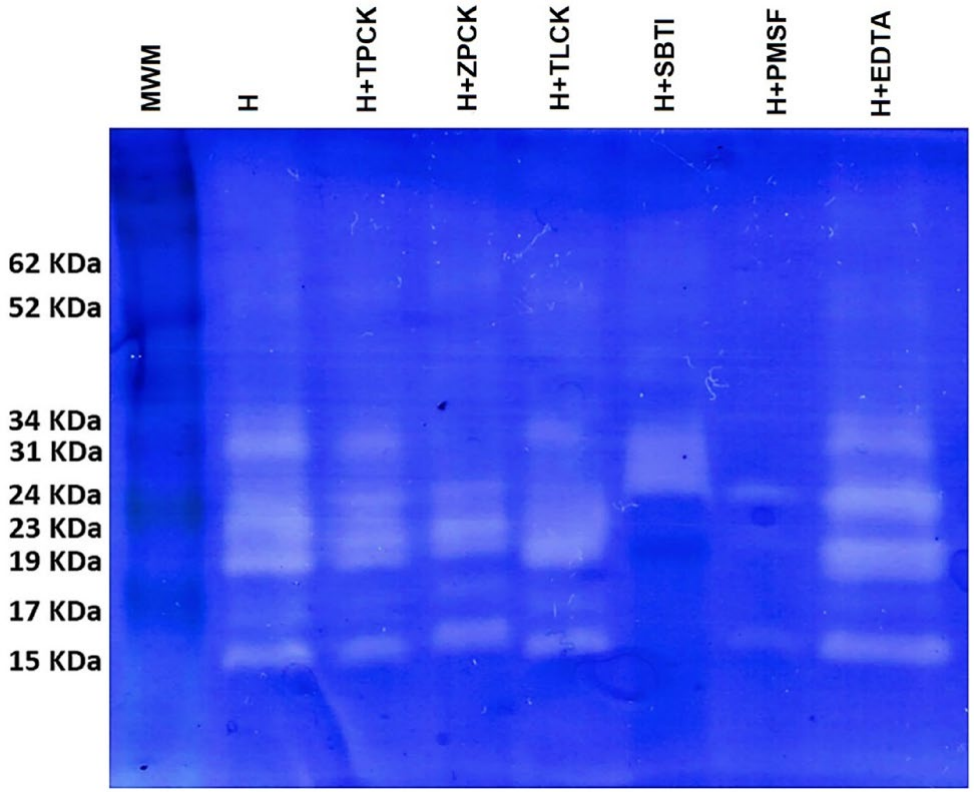
Cropped image from inhibition zimogram of alkaline protease activity in digestive tract extract from juvenile pikeperch at 45 DPF. All samples were analyzed individually; the figure shows a representative result. Samples by lane and from left to right: MWM: Molecular Weight Marker, H: homogenate, H + TPCK (chymotrypsin inhibitor), H + ZPCK (chymotrypsin inhibitor), H + TLCK (trypsin inhibitor), H + SBTI, H + PMSF and H + EDTA. Figures shows the molecular weight of bands with proteolytic activity. Original gels are presented in the supplementary Fig. S1.

Inhibition zimography revealed the presence of nine bands with proteolytic activity in the digestive tract of juvenile pikeperch. All of them were serine proteases and independent of Ca^2+^, as could be seen in lanes 6, 7, and 8. The more significant bands correspond to 31, 24, 23, 19 and 15 KDa. The higher molecular weight bands, 62 and 52 KDa, and the band with 23 KDa showed a weak inhibition by both trypsin and chymotrypsin inhibitors. The band of 34 KDa was inhibited less than 15% by both trypsin and chymotrypsin inhibitors. Moreover, bands with intermediate molecular weight 31 and 24 KDa presented strong inhibition (more than 50%) by the action of TPCK, ZPCK and TLCK. Finally, three bands with low molecular weights, 19, 17 and 15 KDa, were well identified as proteases with chymotrypsin activity (Fig. 3).

**Figure 4:**
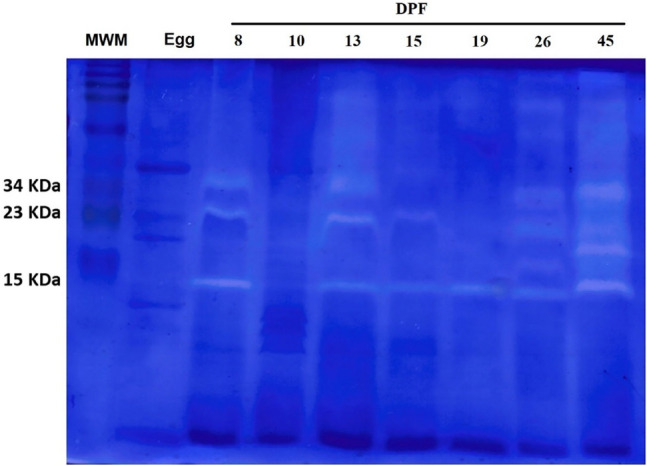
Cropped image of model zimogram of the alkaline proteolytic activity during ontogeny from egg to 21 DPH and digestive tract extract of juvenile pikeperch at 40 DPH. All samples were analyzed individually; the figure shows a representative result. Samples by lane and from left to right: MWM (Molecular Weight Marker), Egg, 8, 10, 13, 15, 19, 26 and 45 DPF. Figure shows the molecular weight of bands with proteolytic activity. DPF: day post fertilization. Original gels are presented in the supplementary Fig. S2.

Zimography results during the ontogeny revealed that in the egg stage and in larvae at 10 DPF (133.5 dd) no digestive enzyme activity could be detected by this technique. The difference between zimography results from digestive enzyme activity and those of total proteolytic activities especially in the egg stage and larvae at 10 DPF (133.5 dd), could be due to the presence of other proteases of non-digestive origin. In homogenates from larvae at 8 DPF (108 dd), three bands were observed, with 34, 23 and 15 KDa. These bands were also detected in larvae at 13 DPF (189 dd), but its activity began to diminish as they grew, finding in larvae at 19 DPF (275.5 dd) just the band with the lower molecular weight. At 26 DPF (378.9 dd) most of the bands that were presented at 45 DPF (777 dd) began to be detected (Fig. 4).

## Discussion

The ontogenetic development of the digestive system in fish larvae provides valuable information and understanding of fish digestive physiology for diet optimization in mass marine fish larviculture^16–18^. Among fish species, the digestive enzymes activity varies in species, age and type of diet^15,19^.

The maturation of the digestive system gradually progresses with larval development. In pikeperch, the functional organs of the newly hatched larvae developed over a relatively short period, less than 15 DPF (211.5 dd) and the inflection points in body proportion changes in pikeperch occurred when the body length was between 9.0 and 12.5 mm. The importance of morphological modifications occurred during the early life periods of pikeperch, showing that these changes coincide with the functional needs throughout the early life period and adaptation to adult life style. In the stage of change from endogenous to exogenous feeding, fish larvae are highly sensitive. Sufficient quantities of food with highest ingestion rate provided enough assimilation to guarantee metabolism and survival during ontogeny^20^. Success of aquaculture in terms of healthy cultured stock depends on highly nutritious quality diet that is readily accepted and digested. The survival rate of pikeperch larvae was high in this study, however, the use of live feed enrichments due to the added nutritional value to pikeperch larvae feed may help the fast growth rate and survival. So future work is recommended to improve the pikeperch larval growth and survival with the modification of the current feeding scheme.

In fish species the successful food consumption depends on the availability of appropriate food items, a developed digestive system and enzyme activities, and the capacity for locomotion^3^. In this study, the mouth was not yet open and the jaw was not yet formed in the endogenous feeding stage. The preflexion stage coincided with the first exogenous feeding at 5 DPH (10 DPF, 133.5 dd) and at the initial of this stage, the depletion of the yolk sac and oil globule were observed along with the endo-exogenous feeding period. According to Ostaszewska et al.^13^ the yolk sac material absorption is accompanied by an intense development of digestive system and the macroscopic yolk sac resorption initiated on the 120 dd (fed with *Artemia* nauplii and formulated diets). The newly hatched larvae of fish soon after yolk absorption starts exogenous feeding, but they have very small mouth size (less than 0.1 mm), primitive digestive system and low digestibility^21^. The size of mouth is very important and determines the ability of the larvae to consume different food organisms^22^. So these larvae require small size, easily digestible, and nutrient riched live feed as their diet. Live feed organisms contain all the nutrients such as essential proteins, lipids, carbohydrates, vitamins, minerals, amino acids and fatty acids^21^. Cladocerans serve as an important food for small fish, because of their small size and jerky movement which make them more visible to fish larvae. Rotifers, *Artemia* and copepods are the main live feeds. Rotifers are suitable for the earliest stages of fish growth because of their small size^23^. In this study, the size of *Artemia* and *Daphnia magna* was less than 1 mm which is suitable for capturing and ingestion of pikeperch larvae between 16 DPF (228.5 dd) to 32 DPF (479 dd). Copepods are considered to be superior in terms of nutritional composition as they contain higher levels of highly unsaturated fatty acids^21^, and they can enhance growth and survival of first-feeding fish larvae.

The water temperature affects the activity of all enzymes. The effects of water temperature on fish digestive enzyme activities seem species-specific, of which it directly affects the digestibility and metabolism of nutrients such as proteins and lipids^24,25^. There is an optimal temperature for digestive enzymes to better participate in the biological reaction process. According to the kinetics of enzymatic reaction and the protein properties of digestive enzymes, the enzymatic activity increases with the increase in temperature up to a certain level^25^. In this study, the growth and activity of some digestive enzymes of pikeperch larvae reached the maximum at higher temperatures (777 dd), under light density of 500 lux and fed with live feed organisms. In *Lutjanus malabaricus* the enzymes activity increased at high temperature (30 °C, as carnivorous fish), which is in accordance with our results^26^. So it can be stated that metabolism increases with temperature increases. Also the similar results were observed in *Catla catla* (optimal temperature 18–28 °C, as omnivorous fish), in which low temperature at 10 °C decreased digestive enzyme activities compared to fish held at 25 °C^27^.

The digestive potential of fish is highly variable, changing with species, water temperature, food and feeding history and development stage. According to our results, the alkaline protease activity of pikeperch larvae showed high levels during the ontogeny from hatching to exogenous feeding, and decreased at the end of larval and juvenile stages. During the larval stages, proteins are digested mainly by alkaline proteases such as trypsin and chymotrypsin^19^. Thus, this study showed that pikeperch larvae have trypsin and chymotrypsin since 8 DPF (108 dd). Before the absorption of the yolk sac, trypsin has the highest level of activity in white sea bass (*Atractoscion nobilis*) larvae cultured at 18 °C^28^. This result is in agreement with the observation in turbot (*Scophhthamus maximus*) cultured under fed and fasting condition, which increased the activity of trypsin, chymotrypsin and alkaline phosphatase enzymes before first feeding^29^. In Siberian sturgeon (*Acipenser baerii*) the activity of chymotrypsin increased after the consumption of exogenous food^30^. An increase in alkaline phosphatase activity was also observed in marine fish, sea bream (*Sparus aurata*), during feeding^31^. In wolf cichlid (*Parachromis dovii*) larvae cultured at 25–29 °C (fed with *Artemia* nauplii and formulated feed), trypsin activity reached the highest level during 19 DPF (275.5 dd)^32^. Generally, in this study trypsin activity was observed during 8 DPF (108 dd) to 19 DPF (275.5 dd), then tended to decrease until 45 DPF (777 dd), although a high activity was observed at the 10 DPF (133.5 dd), which is the day of the start of active (exogenous) feeding. The chymotrypsin activity increased similar to that of trypsin during the first 8 (108 dd) to 19 (275.5 dd) DPF in pikeperch larvae. It may be due to the use of a high protein diet, such as zooplanktons, during exogenous feeding. Vijverberg and Frank^33^ determined 71.2% protein, 19.3% lipids and 9.5% carbohydrates in zooplanktons such as cladocerans. In this study, pikeperch larvae were fed with small zooplanktons until 15 DPF (211.5 dd), followed by *Artemia* nauplii and *Daphnia magna* and other natural live feed until 45 DPF (777 dd). Ahmadi et al.^34^ reported that *Artemia* nauplii has a high amount of C18:3n3 fatty acid and in fresh hatched *Artemia* nauplii proximate composition were calculated as protein 37–71%, lipid 12–13%, carbohydrate 11–23% and ash 4–21%. The contents of protein 47.7%, lipid 6.1%, carbohydrate 20% and ash 15.6% were calculated in *Daphnia magna*^35^. So it can be concluded that the proximate composition of food items influence the digestive enzymes of pikeperch. The pepsin activity was observed at 26 DPF (378.9 dd) and showed the maximum level in the juvenile stage. During the early life stage, pancreas enzymes play a key role in digestion due to the poor developed digestive system^36^. Daries et al.^37^ stated that the complete maturation of gastric glands takes place when these are able to secret pepsin and hydrochloric acid. The appearance of pepsin activity in the transition from larval to juvenile stage in fish species such as pikeperch and common pandora (*Pagellus erythrinus*)^10,38^, red snout cichlid (*Petenia splendida*)^39^, red porgy (*Pagrus pagrus*)^37^ and spotted rose snapper (*Lutjanus guttatus*)^40^ were reported. According to the results of this study, live feeds were considered as a food item during the growth of pikeperch larvae. Live feed organisms facilitate the process of digestion and assimilation by autolysis and by providing their digestive enzymes to fish larvae^41^.

It seems that fish larvae adapt their functional metabolism to nutrients in their environment through enzyme secretion^37^. Therefore, the increase in pepsin activity is associated with a sudden decrease in trypsin and chymotrypsin activity after the yolk sac absorption and the start of exogenous feeding. In other words, the transfer of enzyme activity from alkaline proteases (trypsin and chymotrypsin) to acidic proteases (pepsin) was observed during the growth and development of pikeperch larvae, which can be related to physiological changes that occur during larval development, such as increased protein ingestion, the appearance of hormones or other enzymes, although a type of genetic programming can not be discarded^19,42^.

The α-amylase activity in pikeperch decreased during the larval stages and it increased at 15 DPF (211.5 dd) and in the juvenile stage, which can be related to pikeperch prey during the first stages of exogenous feeding and in larvae-juvenile transition. Castro-Ruiz et al.^6^ suggested that α-amylase activity decreased during larval period in tiger shovelnose catfish (*Pseudoplatystoma punctifer*) larvae cultured at 27.8 ± 0.7 °C (fed with *Artemia* nauplii and formulated feed). In agreement with Pradhan et al.^5^ on butter catfish (*Ompok bimaculatus*) larvae reared at 27.0 ± 1.1 °C (fed with *Artemia* nauplii and zooplankton), and Frías-Quintana et al.^43^ on tropical gar *(Atractosteus tropicus*) larvae cultured at 29.0 ± 1.0 °C (fed with *Artemia* nauplii and trout feed), α-amylase showed low activity at hatching, and it increased gently after exogenous feeding. One of the reasons for digestive enzyme changes during fish ontogeny can be due to larval feeding behaviour and chemical composition of food items^44^. The enzyme activity in fish was closely related to the diet^45,46^ and diet affects the enzyme activity especially at the last stage of larval development when the larvae adapted well to the diet^47^. Hence, it was suggested that different patterns of α-amylase activity during the ontogenesis between species, could be linked to their digestive physiology differences or feeding habits^6,48^. In current study, pikeperch larvae received no food until absorbing the yolk sac, so the presence of amylase at this stage indicates that it is synthesized during the early stages of larval development even in the absence of food. In general, this feature is a genetic programming in fish larvae to digest carbohydrates after the early larval stages^49^.

The study of lipase activity during larvae ontogeny allows to have a clue about the utilization of dietary lipids^6,50^. Based on the current study, an early lipase activity was detected after hatching and the levels of this enzyme in juveniles were significantly higher than those found in larvae. The lipase activity after hatching refers to yolk lipid catabolism as an energy source for larval development before exogenous feeding^51^. The high amount of lipase activity at juvenile stage of pikeperch can be related to the amount of lipid contents in diets, or changes in the nutritional requirements, which are reflected in the growth rate, or the acquisition of full digestive capacities of this species^6^. According to Oozeki and Bailey^51^, the lipase activity divided to two parts: one part is related to yolk sac absorption and the second is related to digestion of exogenous lipids. Since pikeperch larvae received no food until absorbing the yolk sac, so the presence of lipase at this stage can be categorized as the first type. The digestive organs generally increase in both volume and surface area as the larvae grew. Similar to other fish larvae, it seems that the pikeperch larvae would preferably rely on dietary lipids to meet their energy requirements. The increase in volumetric capacity of digestive tract during development and lipase activity increases has been shown for some fishes such as Soldatov's catfish (*Silurus soldatovi*)^52^, striped catfish (*Pangasianodon hypophthalmus*)^53^ and butter catfish^5^.

To our knowledge, this is the first study that has done a characterization of pikeperch trypsin and chymotrypsin-like activities during the ontogeny. In this sense, the results showed the presence of three proteases of 34, 23 and 15 KDa whose activity was reduced during the early development, being only detected the 15 KDa protease at 19 DPF (275.5 dd). This suggests that a change in digestive protease activity was taking part during this developmental stage, as at 26 DPF (378.9 dd) most of the proteases found in juveniles at 45 DPF (777 dd) were found. Accordingly, a different proteases band pattern was found in seabass fingerlings reared at 25 ± 0.5 °C (fed with high starch or lipid diets) compared with juveniles^54,55^. Moreover, in juveniles 3 proteases with low molecular weights (19, 17 and 15 KDa) had been identified as chymotrypsin-like activities as was found in other species, like seabream reared at 22 °C (fed with high dietary carbohydrate inclusion by both protein and lipid replacement) and in sea bass fingerlings reared at 21–22 °C, where the dietary protein sparing effect by lipids and carbohydrates were studied^54,56^. Unlike in those species, no trypsin-like activities were clearly identified since 31 and 24 KDa proteases presented strong inhibition by both trypsin and chymotrypsin inhibitors, whereas for 62, 52 and 23 KDa presented weak inhibition. This dual inhibition could be related with the concentration of each inhibitor. In this sense, further studies are needed to characterize trypsin and chymotrypsin activities, since an imbalance in the trypsin/chymotrypsin rate can lead to an impairment in the availability of amino acids and therefore in nutrient absorption affecting fish growth^57,58^.

## Conclusion

In conclusion, our study shows that temperature is likely one of the important physical environmental factors affecting the growth of pikeperch and may improve the ontogeny and maturation of the digestive enzymes. The pikeperch larvae reared under higher temperature presented a better growth at the juvenile stage, which might be due to higher digestion rates and better food efficiency. Pikeperch larvae showed the low lipase enzyme activity compared to other enzymes, so it can be suggested that live feeds with low lipid levels are more suitable than the diets containing high lipid level. Based on our results, pikeperch larvae have early capability to digest nutrient-dense diet that is high in protein. So, differences in the enzymatic activity are related to type of feeding habits of the studied fish and also high activities of digestive enzymes after the exogenous feeding reflected the gradual maturation of the digestive system.

## Materials and methods

### Larval and juvenile rearing

Pikeperch larvae were obtained by spontaneous spawning of pikeperch broodstock held at controlled conditions under temperature of 13.9 ± 0.4 °C. The wild broodstock were captured from the Aras dam reservoir in northwest Iran and transported to the Dr. Yousefpour Marine Fishes Restocking and Genetic Conservation Center (Siahkal, Guilan, Iran). The caught female and male fish were 4–5 years old and the body weight was 1.1 ± 0.1 kg.

Before the spawning*,* 44 females and 48 males were separately acclimated in twelve concrete ponds (1.8 × 1.8 × 0.5 m) for 5 to 7 days under identical conditions and without feeding. For hormonal induction, fourteen females and sixteen males were held in each rectangular concrete tank (13.0 × 3.1 × 1.1 m) with an artificial spawning nest (50 × 50 cm)^59^ for each pair. The type of spawning nest was artificial turf. The female spawning was induced by injection of 200 IU human chorionic gonadotropin kg^−1^ body weight, whereas male fish received no injections because they were ready to spawn. The pikeperch pairs spawned on artificial spawning nests (for females spawning occured at 80–85 h after injection).

### Larval rearing conditions

The spawned adhesive eggs attached to the nests were then transferred to the circular concrete tanks. The size of the tank was 180 × 50 cm; 1270 L water volume. Hatching occurred at 3nd–5th DPF (day-post fertilization). After hatching the eggs, artificial spawning nests were taken away from the circular concrete tank to supply enough space for the growth and movement of larvae. The mean larval density in each circular concrete tank was 70 ind. l^−1^. The light intensity was 500 lux^60^, and larvae were exposed in natural photoperiod of 12L:12D. The air blowers provided enough oxygen for larvae. The water flow was maintained at 0.3–0.5 L min^−1^. Water exchange by daily siphoning was carried out to remove any waste matter from the bottom of the tanks.

The water quality characteristics were recorded daily in all raring tanks at the same time from 11:00 to 12:00 A.M. During larval culture period, the water physico-chemical characters were: temperature 18.7 ± 0.3 °C, dissolved oxygen 7.2 ± 0.6 mg l^−1^, ammonia nitrogen 0.03 mg l^−1^ and pH 7.8 ± 0.2. Rotifers, cyclopoid copepods, copepod nauplii, or some small cladocerans were mainly used as first prey for the 10 DPF larvae^61,62^. It could be noted that after absorption of yolk sac the pikeperch larvae are very sensitive to starvation due to high metabolic and anabolic demands^11^. The larvae feed were composed of *Artemia* nauplii and then with *Daphnia magna* at the 16 DPF, of which density was kept at about 30 ind. ml^−1^, three times per day (at 8.00; 13.00; and 18.00). The small zooplanktons were obtained from a stock produced in an earthen pond (using caw manure as an organic fertilizer for microalgae growth on which the zooplankton feed)^63^. The *Artemia* nauplii was hatched based on the standard procedure^64^. After 16–24 h, about 70% of the cysts at 29 °C were hatched and fed to the larvae. From 20 DPF to the juvenile stage, larvae were reared in three earthen ponds under identical conditions including water exchange (5–20% every day), feeding (natural zooplankton such as rotifers, caldocerans and copepods)^65^, water temperature (21.5 ± 2.5 °C), water ammonia nitrogen (controlled under 0.03 mg l^−1^) and natural photoperiod. Pond size was 4 ha and larval density was 400 × 10^3^ larva ha^−1^ in the earthen pond.

### Sampling procedure

During the experiment, random samples of eggs before hatching (3nd-5th DPF (day-post fertilization) and daily samples of larvae were collected randomly from each tank (from 6 to 20 DPF) and pond (from 20 DPF to the juvenile stage) at the same time of day. Larvae were killed with an overdose of clove powder extract and snap frozen in liquid nitrogen. All samples were stored at − 80 °C until further analyses.

Pikeperch survival rate during developmental stages was calculated by the following formula^66^.

$$ {\text{Survival rate }}\left( \% \right)\, = \,{\text{Final fish number}}/{\text{Initial fish number}}\, \times \,{1}00. $$

### Biochemical analyses

#### Sample preparation

Sample homogenization, pepsin, total alkaline protease activities, trypsin and chymotrypsin characterization were performed according to García Meilán et al.^56,67^. Briefly, eggs, whole larvae from day 8 to 15, larvae at 19 and 26 DPF without head and tail, and individually digestive tract without gallbladder in juveniles at 45 DPF were weighed and buffer solution (Tris–HCl, 50 mM, pH 7.5) was added to a final concentration of 200 mg ml^−1^. Samples were then homogenized using rapid vibration (6500 rpm; 3 × 20 s with three breaks of 20 s; 4 °C) in a Precellys Evolution® Homogenaizer combined with Cryolys^®^ as a cooling system (Bertin Technologies, France). Next, homogenates were centrifuged for 15 min (2400 rpm; 4 °C; Eppendorf, 5418R) and supernatants were stored at − 80 °C for future analysis. The 4 different homogenates were used from larvae at the different stages (DPF) and 9 individual samples for juveniles.

#### Digestive enzyme activity

For determination of acid protease activity, homogenized samples were reacted with 50 mM glycine–HCl buffer containing 1% bovine hemoglobin, pH 2 at 25 °C, whereas for total alkaline protease activity (TPA), samples were reacted with 50 mM Tris–HCl buffer containing 1% casein, pH 9.0 at 25 °C. After 60 min, trichloroacetic acid 10% was used for the reaction stop. The samples from both analyses were kept at 4 °C for an hour and then centrifuged (7500 rpm, 5 min, 4 °C). Individual blanks were established for each sample. Supernatant absorbance was measured at 280 nm (Infinite 200 PRO, Grödig, Tecan, Austria). Pepsin from porcine gastric mucosa (Sigma Aldrich, Madrid, Spain, 3440 U/mg solid) was used as standard and acid protease activity was measured as BAEE units. Bovine trypsin (Sigma Aldrich, Madrid, Spain, 12,100 BAEE U/mg protein, NC-IUB, 1979) was used as the standard.

The protein concentration in homogenate samples was measured according to Bradford^68^ method using bovine serum albumin as a standard.

Lipase and α-amylase enzymes activity was determined according to the kit manufacturer’s recommendation (Spinreact, Sant Esteve d’en Bas, Girona, Spain). For lipase, methylresorufin formation was measured at 580 nm. For α-amylase, the rate of 2-cloro-4-nitrophenol formation at 405 nm was determined. Activities of both enzymes were reported as mU per mg of protein. Both tests were conducted at 25 ± 0.5 °C using a microplate scanning spectrophotometer (Tecan Infinite 200 PRO, Grödig, Tecan, Austria).

For digestive activities, 4 pools of samples from egg to 26 DPF stage and 9 individual samples for juveniles (45 DPF) were analysed.

#### Zimogram

Trypsin and chymotrypsin-like activities were characterized by zimography according to Santigosa et al.^69^. Proteolytic characterization was done by the combination of the homogenate with water or the corresponding inhibition solution for 45 min at a ratio of 1:1. Inhibition solutions selected were: tosyllysyl chloromethyl ketone (TLCK; 10 mM in HCl 1 mM) as trypsin-like activity inhibitor, tosyl phenylalanyl chloromethyl ketone (TPCK; 10 mM in methanol) and carbo benzoxyphenylalanyl chloromethyl ketone (ZPCK; 10 mM in dioxane) as chymotrypsin-like activity modifiers, SBTI (250 µM in H_2_Od) and PMSF (100 mM in 2-isopropanol) as total serine protease activity inhibitor and EDTA (0.5 in H2Od) to determine protease dependence on divalent cations, according to Alarcon et al.^70^.

Samples were loaded in 12% polyacrylamide gels (10 × 10.5 × 0.1 cm), pure trypsin and albumin were used as controls and a commercial weight marker RPN 800 (Amersham, GE Healthcare, UK, 12,000–225,000 Da) was used to determine the molecular weight of protease active fractions from the homogenized samples. Electrophoresis was performed at a constant current of 15 mA per gel for 100 min (Biorad 4 °C). The gels were incubated under agitation with TrisHCl buffer containing 2% casein, pH 8.2 for 30 min at 4 °C, and then it was shaken for 90 min at room temperature. Gels were washed and stained in a methanol:acetic acid:water solution (40:40:10) with 0.1% BBC R-250 (Coomassie Brilliant Blue R-250) for 15–20 min. After that, the same solution without colorant was used for destaining for 5–10 min and agitation was applied during both procedures.

Trypsin and chymotrypsin-like activities were characterized by 3 inhibition zimograms for juvenile stage (Supplementary Fig. S1). Protease activity during pikeperch ontogeny was detected by running 4 zimography gels, in which every gels contains one homogenate from egg to juvenile stages (Suplemmentary Fig. S2).

#### Signifcance statement

This work contains important studies on pancreatic and proteolitic enzymes for the evaluation of functional ontogeny of digestive enzyme of pikeperch from hatching to 45 days post fertilization (DPF) under culture condition. Inhibition zimography reveals the proteolytic activity in the digestive tract of juvenile pikeperch. All digestive enzymes were detected at egg, except pepsin. Also, larvae had the ability to digest diet that was high in protein. Despite this, they showed low lipase activity compared to other enzymes. In summary, larvae possess a functional digestive system with high enzyme activities after the exogenous feeding that indicated the gradual development of the digestive system.

#### Statistical analyses

Data were analyzed using IBM SPSS Statistics v.25 (Armonk, USA) and were presented as means ± standard error of the means (SEM). The normal distribution was analyzed using the Shapiro–Wilk test and homogeneity of the variances (homoscedasticity) was assessed with Levene’s test. If normal distribution and/or homoscedasticity were not found, data were transformed logarithmically. Significant differences were tested by One-way analysis of variance (ANOVA) and the post-hoc Tukey HSD. If necessary, the nonparametric Kruskal Wallis test and the post-hoc Games-Howell were used. Statistical differences were considered significant when *p* < 0.05. Zimogram inhibition results were analyzed using Quantity One 1-D Analysis Software 4.6.6 (Bio-Rad Laboratories, Inc, Hercules, California, USA).

### Ethics approval

All the experimental animal procedures involved in this study were approved by the animal care and welfare of University of Guilan and followed the experimental basic principles by the ARRIVE guidelines.

All the reporting in the manuscript follows the recommendations in the ARRIVE guidelines for Reporting Animal Research. No distress or suffering was produced by the procedures used to perform this study.

### Supplementary Information

Supplementary Figures.

## Data Availability

The data that support the findings of this study are available from the corresponding author upon reasonable request.
