# Supplementary material for: Ontogeny of the digestive enzyme activity of the pikeperch (Sander lucioperca) under culture condition
Source: Sci Rep. 2023 Nov 13;13:19739. doi: 10.1038/s41598-023-43845-w (PMC10643626; doi:10.1038/s41598-023-43845-w)
Supplement: Supplementary file 1 — Supplementary Figures. [file 41598_2023_43845_MOESM1_ESM.pdf]

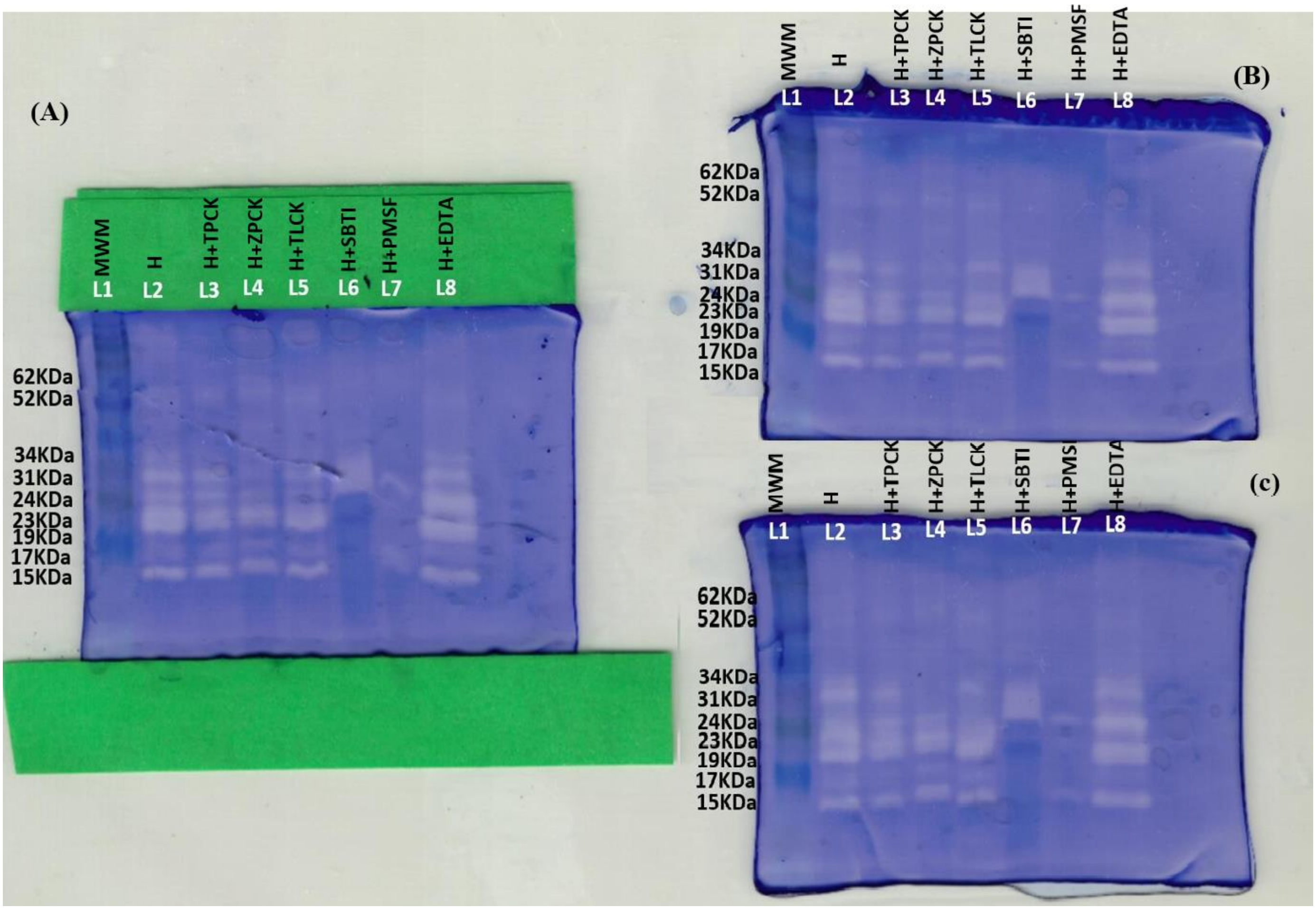

Figure S1. Original inhibition zimograms of alkaline protease activity in digestive tract extract from 3 samples of juvenile pikeperch at 40 DPH (days-post hatching) (A, B & C). Figure shows the molecular weight of bands with proteolytic activity. L (Lane, L1 to L8 for all three gels are same). MWM (Molecular weight marker), H (homogenate), H+TPCK (chymotrypsin inhibitor), H+ZPCK (chymotrypsin inhibitor), H+TLCK (trypsin inhibitor), H+SBTI, H+PMSF and H+EDTA.

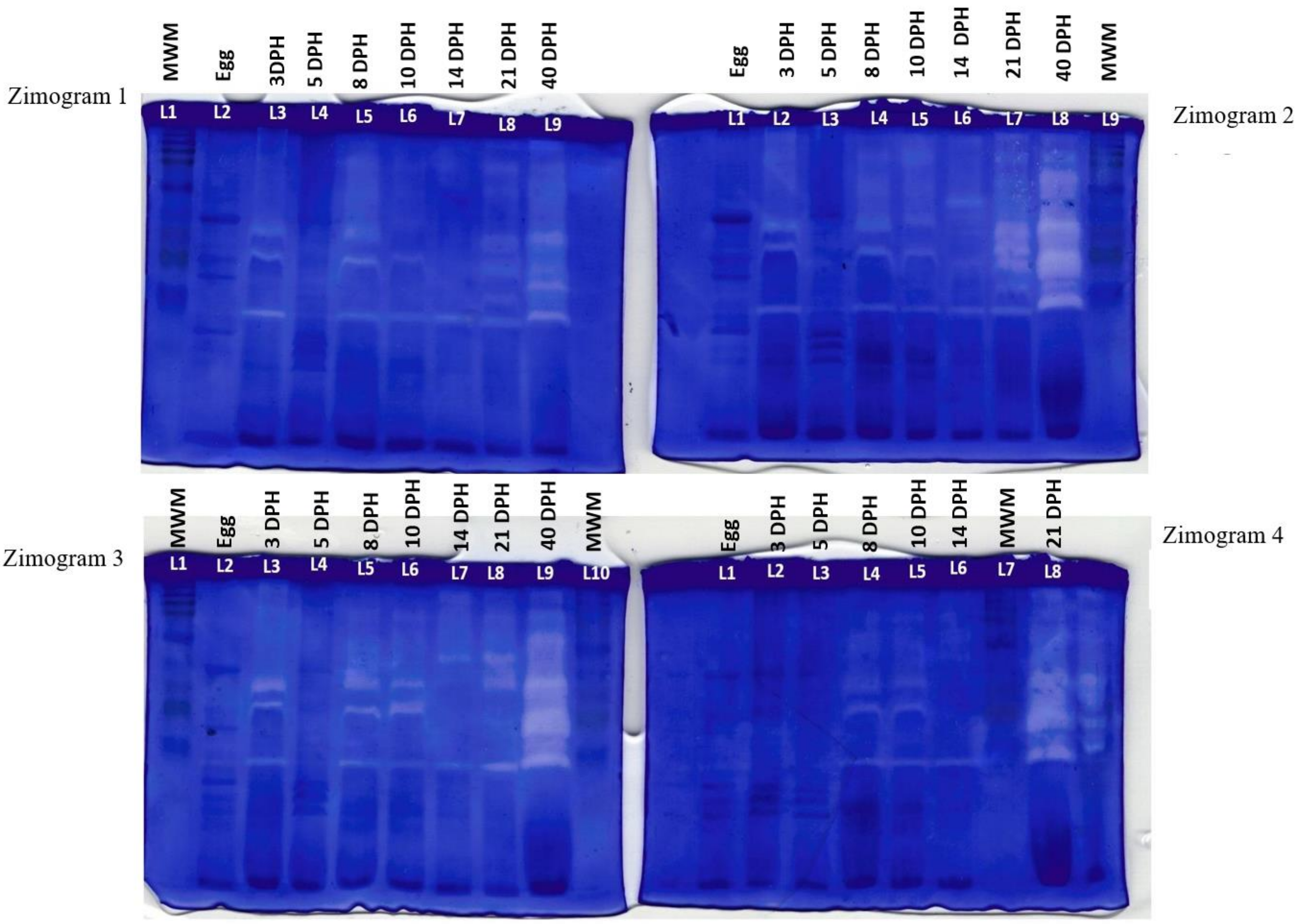

Figure S2. Original zimograms of sample homogenates pools 1, 2, 3 and 4 to characterize alkaline proteolytic activity during ontogeny in larvae from egg to 21 DPH and digestive tract extract ofjuvenile pikeperch at 40 DPH. All samples were analyzed individually; zimogram 1 was chosen as representative result. DPH: days- post hatching; L: Lane.
